# In Vitro Effects of Vanadate Erbium/Silver Oxide (ErVO_4_/AgO) and Vanadate Iron/Silver Oxide (FeVO_4_/AgO) Nanoparticles on the Adult of *Fasciola hepatica*

**DOI:** 10.1002/vms3.70357

**Published:** 2025-04-28

**Authors:** Arbabi Mohsen, Esmaili Monireh, Hooshyar Hossein, Delavari Mahdi, Sobhani Nasab Ali, Nejati Majid

**Affiliations:** ^1^ Department of Parasitology Mycology, School of Medicine Kashan University of Medical Sciences Kashan Iran; ^2^ Physiology Research Center Institute for Basic Sciences Kashan University of Medical Sciences Kashan Iran; ^3^ Anatomical Sciences Research Center Institute for Basic Sciences Kashan University of Medical Sciences Kashan Iran

**Keywords:** *Fasciola hepatica*, nanoparticles, ultrastructure changes, Vanadate erbium/silver oxide (ErVO_4_/AgO), Vanadate iron/silver oxide (FeVO_4_/AgO)

## Abstract

Fascioliasis is a common hepatic parasitic disease that is caused by *Fasciola*, resulting in significant economic losses by reducing production and consigning viscera in animals. Currently, there is little research regarding the impact of chemical compounds on the ultrastructure and motility of adult *F. hepatica*. The present study aims to assess the effect of Vanadate erbium/silver oxide (ErVO_4_/AgO) and Vanadate iron/silver oxide (FeVO_4_/AgO) nanoparticles against liver fluke *F. hepatica*, in vitro assay. *Fasciola hepatica* adult worms were collected from the livers and gallbladders of sheep and goats centrality of Iran. One hundred fresh worms were incubated with each nanoparticle concentration of 4.5–6 mg/mL FeVO_4_/AgO and ErVO_4_/AgO (test, groups) in comparison to triclabendazole 5–20 µg/mL (positive control) and RPMI media culture (negative control) after 12 and 24 hours of treatment. To ensure the reliability of the data, the tests on the sample were performed twice. The effectiveness of these compounds was evaluated by examining parasite movement, reaction to vital stain and changes in the tegument through scanning electron microscopy (SEM) using Fisher statistical tests and logistic regression. Analysis of variance was performed to compare Kaplan–Meier and Cox groups and models to analyse parasite survival. In addition, the anthelmintic efficacy was measured as the mortality rate based on the number of live and dead worms. The mortality ratios show that the anthelmintic activities of the compounds highly relied on time and concentration, as time and concentration increased, increasing the mortality rate. Lethal concentration 50 (LC_50_) of FeVO_4_/AgO and ErVO_4_/AgO are 4, 4.7 and 5 mg/mL at 24 h, respectively. FeVO_4_/AgO showed more lethal effects on *F. hepatica* than on ErVO_4_/AgO and triclabendazole. SEM analysis of treated *F. hepatica* by both nanoparticles at a concentration of 6 mg/mL showed that the tegument surface of fasciola is swollen in some parts, the pores on the tegument surface are completely visible, the sensory papillae are lost, the tegument is severely damaged and the prominent network structure and its vesicles have completely disappeared. *F. hepatica* is more susceptible to the lethal effects of FeVO_4_/AgO and ErVO_4_/AgO nanoparticles. The effectiveness of these compounds depends on the concentration and time of the drug's effect, in such a way that the effectiveness increases with the increase in concentration and time.

## Graphical abstract

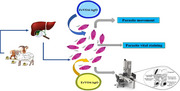

## 1 Introduction

Foodborne diseases (FBDs) are a significant risk to public health, potentially leading to loss of life and impacting socioeconomic status (Chhetri et al. 2021). Among FBDs, fasciolosis is a prominent neglected zoonosis in tropical and subtropical regions; it is primarily caused by the liver flukes *Fasciola hepatica* and *F. gigantica*, posing a major concern. Its zoonotic risk and mode of transmission make fasciolosis a considerable threat to veterinary public health. Besides lower milk production in dairy cows, there are liver contamination, loss of body weight and increased vulnerability to other diseases. This significantly affects veterinary science, especially in sheep and cattle breeding, resulting in substantial economic losses due to liver and carcass condemnations (Prasetyo et al. 2023). Annually, millions of animals and humans suffer from parasitic infections, leading to significant deaths per annum. Research shows more than 180 million individuals are at risk of infection worldwide, while between 35 and 72 million people are thought to be infected with Fasciola species, and this figure is probably on the rise.  Fasciolosis results in significant economic losses for the agricultural industry, estimated at over US$3 billion globally each year (Utrera‐Quintana et al. 2022). In many parts of the world, parasitic diseases are neglected, contributing to a high incidence of fatalities associated with these infections. Various treatment approaches have been employed to control parasites; however, their effectiveness has diminished due to the emergence of resistance among parasites and the adverse effects associated with traditional treatment methods (AlGabbani 2023). Currently, approximately 17 million individuals have been diagnosed with fascioliasis, and it is anticipated that around 91 million more may be infected across various regions globally (Ghanimatdan et al. 2019; Seid and Melese 2018). Annually, the economy of countries suffers direct and indirect damage amounting to US$3 billion due to productivity losses caused by the destruction of infected liver in farmed animals, specifically cattle and sheep, as a result of fascioliasis (Mehmood et al. 2017). In economic contexts, losses can result from multiple contributing factors, including costs of medicines, damage to meat quality, inferior wool production, diminished milk yield, lower calf birth weights, a decline in average growth rates and increased susceptibility to secondary infections (Calvani et al. 2020; Muhammad et al. 2019). The most important ruminant parasite globally responsible for zoonotic fascioliasis is *F. hepatic* (Howell et al. 2020), which impacts approximately 700 million herbivorous domestic animals (Zhang et al. 2019). Fascioliasis is characterized by inflammation of the liver and bile ducts, which can be chronic, sub‐acute or acute in nature. In both acute and chronic cases, the migration of juvenile worms into the liver parenchyma results in liver damage, haemorrhaging and dilation of the bile ducts. Dysfunction of the liver, along with cirrhosis, can result in submandibular oedema, subsequently leading to anaemia and loss of appetite. In severe instances, these complications may culminate in systemic intoxication and potentially fatal outcomes over prolonged durations (Valero et al. 2016; Portugaliza et al. 2019). The most effective approach for managing animal parasitic diseases continues to be the utilization of chemical antiparasitic medications. These products are crucial for ensuring the safety of animal health; however, many traditional formulations exhibit poor absorption owing to their insolubility, as well as low bioavailability and short half‐lives. Additionally, certain antiparasitic medications are susceptible to enzymatic degradation or inactivation within the animal's system, resulting in a significant first‐pass effect. It also indicates that there is inadequate penetration of the biological membrane barrier between tissue and cells, leading to diminished bioavailability and a less‐than‐expected therapeutic effect. Furthermore, the prolonged life cycles of parasites necessitate frequent dosing for effective treatment over extended periods. This repeated administration can cause stress in animals, increase the labour intensity for farmers and contribute to the development of drug resistance (Sun et al. 2019; Lu et al. 2017; Babita et al. 2018). Chemotherapy continues to be the primary method for controlling fasciolosis in endemic regions. However, treatment remains a significant technical and legal obstacle. The limited availability of flukicide drugs poses a significant challenge for certain productive sectors (Castro‐Hermida et al. 2021).

**Summary**

- Although TCBZ is the only effective antiparasitic drug available now for fascioliasis, new drugs are urgently needed.
- Anthelmintic activities of FeVO_4_/AgO and ErVO_4_/AgO were examined on the ultrastructure of adult *F. hepatica*.
- FeVO_4_/AgO and ErVO_4_/AgO nanoparticles caused significant degradation to the parasite tegument and killed worms.
- The anthelmintic activity of the studied compounds depends on time and concentration.
- Using the SEM method, these compounds were shown to have a lethal effect on the parasite.

In humans, the treatment of fascioliasis is based on the use of anthelmintics to eliminate flukes, followed by symptomatic treatment to relieve abdominal pain, and possibly the use of antispasmodics to treat biliary colic. This condition can arise from the accumulation of dead or dying flukes within the bile ducts, potentially causing obstruction of the drainage system. At present, triclabendazole (TCBZ) is the only pharmaceutical agent recognized by the World Health Organization for its efficacy against infections caused by both early immature and adult stages of *F. hepatica*. However, the prevalence of resistance to this medication significantly undermines the management of fluke infestations in both livestock and humans. Furthermore, there have been reports indicating a decline in the effectiveness of these chemical anthelmintics, potentially due to the emergence of resistant strains. The precise mechanism by which TCBZ operates and the factors contributing to the parasite's resistance remain unclear (Gandhi et al. 2019; Luis Marcos et al. 2021).

Nanoparticles (NPs), with their capacity to overcome insufficiencies in bioavailability, lack of cellular permeability, no particular distribution, minimizing toxicity to the host and a quick disposal of antiparasitics drugs from the body, are novel drug carriers that might offer a successful way for treating parasitic infections. In recent years, various types of ideal nanocarriers have been developed for the delivery of antiparasitic drugs and to enhance drug stability. NPs, including inorganic components, have attracted people's attention to antiparasitic drug delivery with the rapid development of nanotechnology and human demand for parasitic disease treatment (Tiwari et al. 2023).

The distinctive characteristics of NPs present benefits that surpass those of alternative treatment options, particularly due to their non‐toxic nature and the absence of resistance from parasites. Various conventional and molecular techniques employed in the preparation of NPs have demonstrated efficacy in eliminating parasites or inhibiting their growth, and these NPs can also serve as diagnostic tools for parasitic infections. Gold and silver NPs have been the focus of extensive research; however, other NPs, including those made from iron, nickel, platinum and copper, have not been studied as thoroughly in terms of their potential effectiveness in controlling diseases in both animals and humans. In recent decades, nanotechnology has represented a significant advancement within the medical and pharmaceutical sectors, particularly concerning the application of nanotools for the treatment and management of infectious diseases. Nevertheless, further investigation is essential to elucidate the mechanisms by which NPs operate, which is crucial for the creation of safe diagnostic and therapeutic options (Bajwa et al. 2022). Research has been conducted in this area, validating the anthelmintic properties of zinc oxide NPs (ZnO) against the trematode *Gigantocotyle explanatum* (Khan et al. 2015). In a certain study, the anthelmintic properties of zinc oxide (ZnO) and iron oxide (FeO) NPs against *Toxocara vitulorum* were shown (Dorostkar et al. 2017).

In a similar study, the antihelminthic effects of silver and NPs against *Haemonchus contortus* and *Raillietina* helminths were demonstrated (Tomar and Preet 2017; Kar et al. 2014). Erbium vanadate/silver oxide and iron vanadium/silver oxide are the two nanocomposites used in the present study, both of which use silver NPs due to their special properties. Silver oxide NPs (AgO) have effective antibacterial effects, including on *Staphylococcus aureus*. Compounds based on this NP have various applications: including in water treatment, soil sterilization for growing greenhouse vegetables and the production of medical products (Shen et al. 2017).

To the best of our knowledge, no reports are available on the in vitro assessment of the efficacy of Vanadate erbium/silver oxide (ErVO_4_/AgO) and Vanadate iron/silver oxide (FeVO_4_/AgO) NPs against the adult of *Fasciola hepatica*.

## 2 Materials and Methods

### 2.1 Materials and Physical Measurements

All chemicals employed in this procedure were of analytical grade and utilized as received, without any additional purification. X‐ray diffraction (XRD) patterns were obtained using a Philips X'PertPro X‐ray diffractometer, employing Ni‐filtered Cu Kα radiation within a scan range of 10<2θ<80. Scanning electron microscopy (SEM) images were captured using a LEO‐1455VP, which is equipped with energy‐dispersive X‐ray spectroscopy.

### 2.2 Synthesis of FeVO_4_

In a standard synthesis protocol, 1 mmol of NH_4_VO_3_ and 1 mmol of Fe (NO_3_)3·9H_2_O were individually dissolved in 40 mL of ethanol while applying ultrasonic agitation and stirring. Subsequently, the iron solution was gradually introduced into the vanadate solution with continuous stirring and ultrasonic treatment. Following this, the resultant mixture was maintained under stirring to remove the solvent and facilitate gel formation at a temperature of 90°C. Ultimately, the resulting product underwent calcination at 500°C for 120 minutes in a conventional furnace within an air atmosphere.

### 2.3 Synthesis of ErVO_4_

In this study, ErVO_4_ NPs were synthesized using the co‐precipitation technique. Initially, ErNO_3_·6H_2_O was dissolved in 30 mL of water, referred to as solution A. Subsequently, solution B was prepared by dissolving 1 mmol of NH_4_VO_3_ in another 30 mL of water. Solution A was then combined with solution B under vigorous and continuous stirring to produce a solution C. The resulting precipitate was washed three times with distilled water, followed by drying and calcination at a temperature of 500°C for 90 minutes.

### 2.4 Synthesis of FeVO_4_/AgO and ErVO_4_/AgO Nanoparticle

**FIGURE 1:**
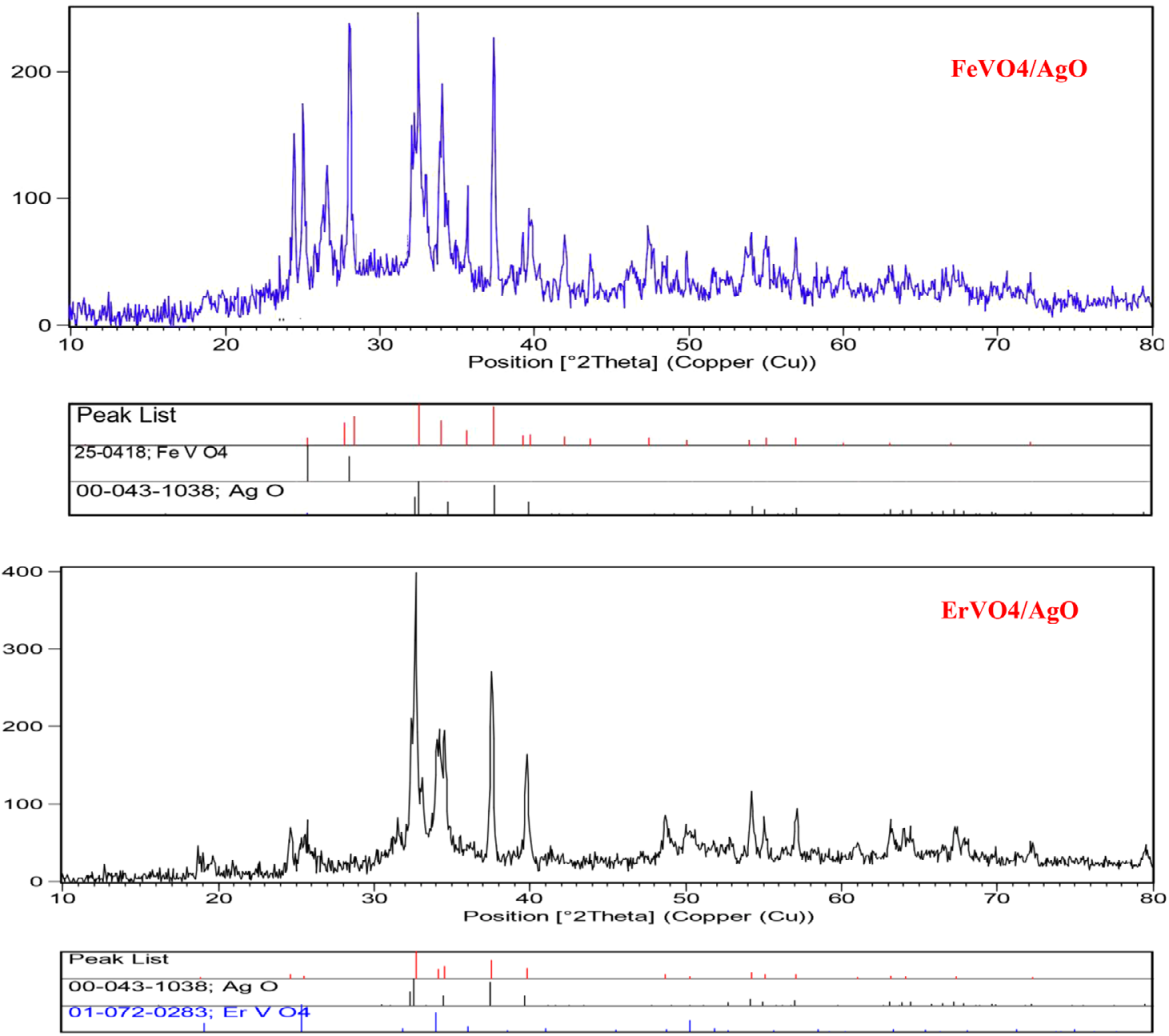
Structure of FeVO_4_/AgO and ErVO_4_/AgO NPs by XRD.

To synthesize AgO NP, 1 g of silver nitrate was dissolved in 20 mL of deionized water under ultrasonic irradiation at 220 W and continuous stirring to achieve a clear solution. Subsequently, this solution was added dropwise to the FeVO_4_ and ErVO_4_ solution maintaining constant stirring and ultrasonic conditions. Following this, 1.5 g of potassium persulfate (k2s2o8), a potent oxidizing agent, was added to the mixture at the same ultrasonic irradiation and stirred for 15 minutes. Ultimately, the resulting grey precipitate was filtered, washed three times with distilled water and then dried at 60°C for 24 hours. The XRD pattern aligns perfectly with the diffraction patterns of FeVO_4_/AgO, as indicated by reference card number 25–0418, and silver oxide, referenced by card number 1038–43, both of which exhibit a cubic crystalline structure. The particle size of the nanoparticle crystals was determined to be 13 nm through the application of the Debye‐Scherrer formula. Furthermore, the XRD pattern also corresponds entirely to the diffraction pattern of ErVO_4_/AgO, referenced by card number 72–0283, along with silver oxide, which is identified by reference card number 43–1038, and similarly crystallizes in the cubic phase. The Debye‐Scherrer formula was utilized to calculate the particle size of these nanoparticle crystals, yielding a measurement of 11 nm (Figure 1).

**FIGURE 2:**
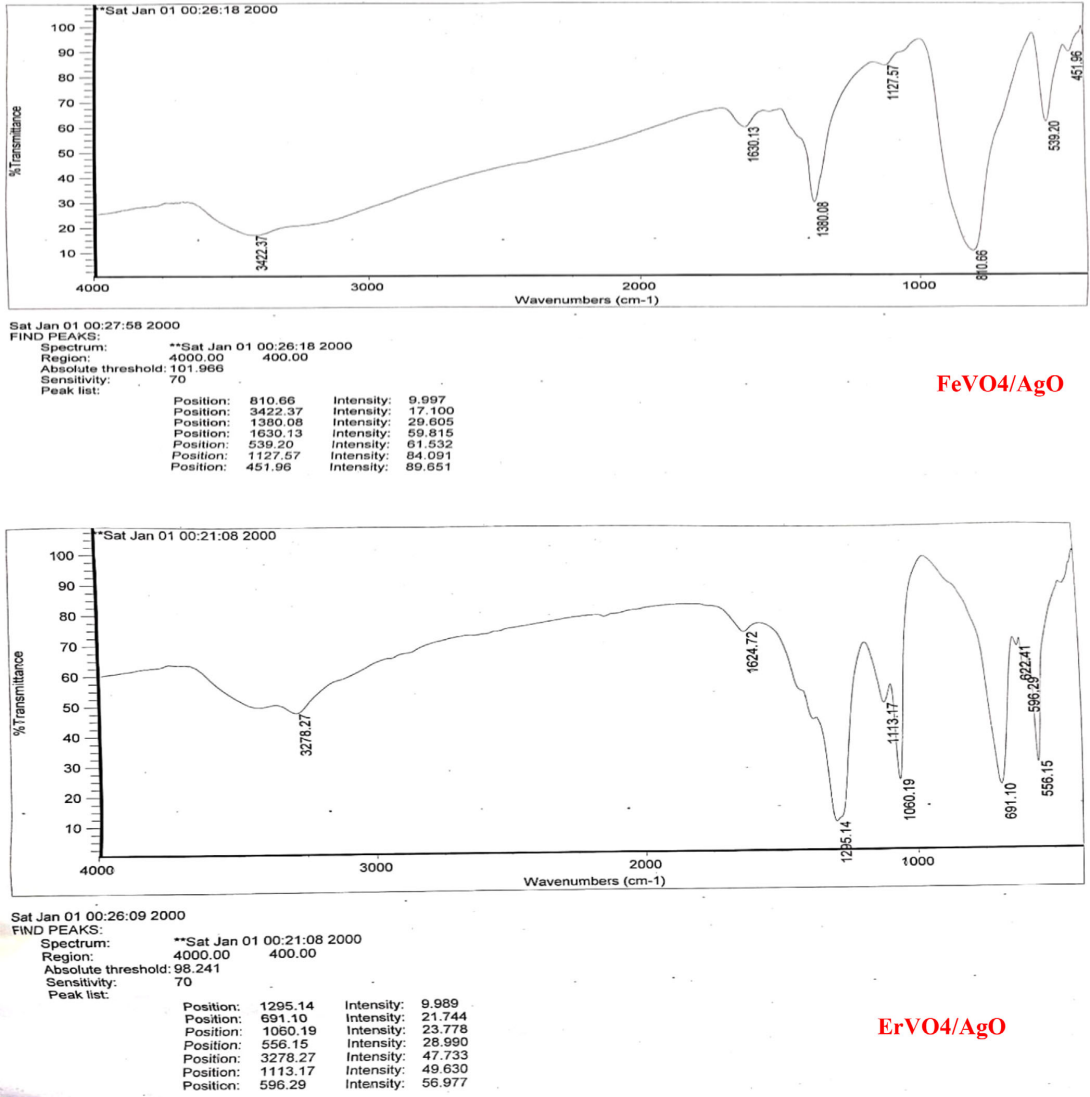
FT‐IR results of FeVO_4_/AgO and ErVO_4_/AgO NPs. SEM images of FeVO_4_/AgO and ErVO_4_/AgO NPs showed that the samples are composed of spherical NPs with a size of ‘**40nm**’ and 20 to 30 nm, respectively (**Figure** **3**).

Figure 2 shows the Fourier transform infrared spectroscopy (FT‐IR) of FeVO_4_/AgO and ErVO_4_/AgO NPs. As it is clear in the spectrum, in the case of FeVO_4_/AgO and ErVO_4_/AgO, the peaks of 3422 and 1630 cm^−1^ as well as 3278 and 1624 cm^−1^ are related to the stretching vibration and bending vibration of water. In the FT‐IR spectrum of the FeVO_4_/AgO and ErVO_4_/AgO sample, the absorption bands at 1451 cm^−1^ indicate Fe‐O, O‐ and Ag‐O, and at 1556 cm^−1^ indicate Er‐O, O‐V‐O and Ag‐O, which confirms the formation of iron vanadate/copper oxide and erbium vanadate/copper oxide nanocomposites, respectively.

### 2.5 Collection of *F. hepatica*

At the Kashan slaughterhouse, data regarding the origins of each animal were recorded. Following the slaughter process, the liver and its associated bile duct underwent a thorough examination during the post‐mortem assessment to detect the presence of liver flukes. Adult *F. hepatica* specimens were isolated from the livers of the slaughtered goats and sheep. Upon arrival at the laboratory, the samples were subjected to three washes with PBS buffer (pH 7–7.3) in preparation for further analysis. Subsequently, they were promptly placed in 6‐well plates containing RPMI‐1640, supplemented with 50 IU/mL of penicillin, 50 IU/mL of streptomycin, 50% v/v of fetal bovine serum (FBS) and 2% sheep red blood cells for various treatments. Only those worms exhibiting a normal tegument and demonstrating motility upon visual inspection were selected for further study.

### 2.6 Evaluation Efficacy of NPs Against *F. hepatica* Adults

For this experiment, *F. hepatica* adults were collected from the liver and gallbladder of sheep and goats diagnosed with fasciolosis on post‐mortem examination. To evaluate the antiparasitic activities, five adult worms were transferred to 6‐well plates, and 4 mL of the NPs was added in each well. The assays were performed in triplicate. The positive and negative controls consisted of TCBZ and RPMI culture media, respectively (Fairweather et al. 2012).

The plates were incubated in a 5% CO_2_ incubator for 2 days at 37°C. Adults’ counts were performed once every hour during the 3‐hour light exposure using a stereoscopic microscope at 2× magnification. The efficacy, expressed as a percentage, was estimated for each combination using the following formula:

$$\begin{array}{ccc} & & \mathrm{Efficacy}\;\;\left(\%\right)\\ & & \;=\frac{\mathrm{mean}\;\mathrm{negative}\;\mathrm{control}-\;\mathrm{mean}\;\mathrm{of}\;\mathrm{NP}\;\mathrm{treatment}}{\mathrm{mean}\;\mathrm{negative}\;\mathrm{control}}\;\times 100 \end{array}$$

NPs with a statistically significant difference between treatment and control groups with efficacy ≥90% were considered effective (Wood et al. 1995). Analysis of variance at *p* < 0.05.

The study was conducted across four distinct groups. Each treatment involved varying concentrations of experimental groups (NPs: 4.5, 5, 5.5 and 6 mg/mL), a positive control treatment (TCBZ: 5, 10, 15 and 20 µg/mL) and a negative control treatment (RPMI‐1640 media culture alone). To facilitate this, adult liver flukes of *F. hepatica* were transferred into six wells under sterile conditions within a laminar flow cabinet, with each well containing 4 mL of RPMI‐1640 supplemented with 50 IU/mL of penicillin, 50 IU/mL of streptomycin, 50% v/v of FBS and 2% sheep red blood cells. Subsequently, 4 mL of ErVO_4_/AgO, FeVO_4_/AgO and TCBZ was introduced into each well, followed by incubation for 12 and 24 hours at 37°C in a 5% CO_2_ atmosphere.

### 2.7 Motility Examination

The motility time of worms, assessed at 12 and 24 hours following incubation in various concentrations of the treatments and control groups, was determined. The viability of the experiments was evaluated based on specific motility criteria, which included whole body movement, partial body movement, reduced overall movement and complete immobility. Trematodes that exhibited no visible movement were further examined under a light microscope to confirm their state of motion. The motility assay for the control groups was recorded in the absence of experimental compounds. Each worm in the experiments was observed individually at hourly intervals, adhering to the established motility criteria.

### 2.8 SEM Sample Preparation

To assess the ultrastructural changes in the tegument of worms from both the test and control groups, we employed SEM. Initially, the treated and control flukes were fixed using sodium cacodylate buffer (pH: 7.4, 0.2 M) combined with glutaraldehyde in phosphate buffer (2.5% v/v) for 4 hours at 4°C. Following this, the parasites underwent three washes in phosphate buffer (pH 7.4). Subsequently, they were dehydrated through a series of increasing ethanol concentrations (70%, 80%, 95% and 100%) for 30 minutes each. The final dehydration was performed using hexamethyldisilazane, after which the samples were dried in a vacuum oven (Ullah et al. 2017).

Finally, the samples were affixed to stubs, coated with a layer of gold through sputter deposition and subsequently photographed using an SEM (ZEISS—DSM 960A, Germany) at the central laboratory of the Kimiazi Institute in Tehran, Iran.

## 3 Results

### 3.1 Structural Study

**FIGURE 3:**
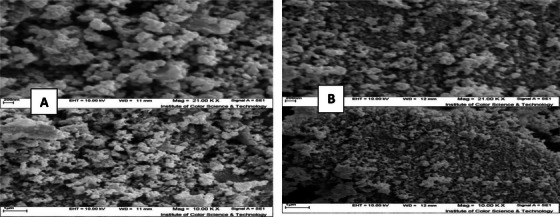
(A) SEM image of FeVO_4_/AgO NPs. (B) SEM image of ErVO_4_/AgO NPs.

**FIGURE 4:**
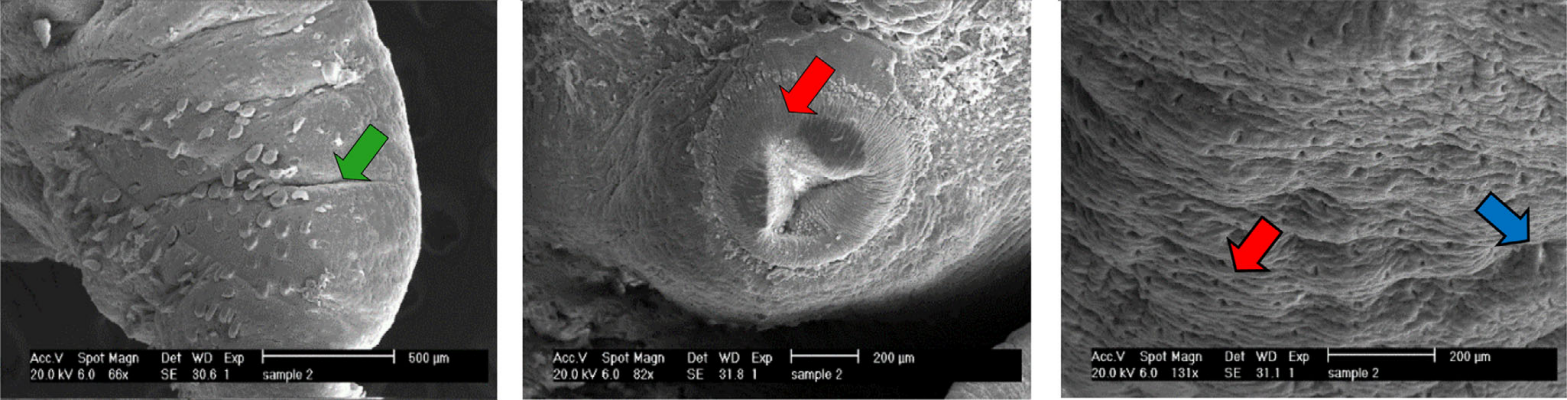
Ultrastructural changes of adult *F. hepatica* worm after exposure to ErVO_4_/AgO NPs with a concentration of 6 µg/mL in 24 hours.

The XRD patterns of FeVO_4_/AgO are presented in Figure 1. This figure illustrates the diffraction peaks with the 2θ = 25.5 (101), 31.58 (111), 32.29 (002) and 37.12 (020) crystal plane of orthorhumbic structure BaFe12O19 [Space group P‐1, JCPDS code 25‐0418] show the as‐synthesized AgO nanostructures, and the diffraction peaks with the 31.6 (110), 32.8 (200) and 40.6 (111) crystal plane of monoclinic structure Sm2Ti2O7 [Space group P21/c, JCPDS code 22‐0472] show the as‐synthesized pure AgO nanostructures. The XRD pattern of FeVO_4_/AgO nano‐hybrid is similar to pure FeVO_4_ and AgO except for absorption intensity and precise position. In Figure 2, the diffraction peaks observed at 2θ = 31.6 (110), 32.8 (200) and 40.6 (111) correspond to the AgO composite within the ErVO_4_ structure. Extensive studies have been conducted by researchers to manipulate the morphology of NPs through the optimization of several parameters, including ultrasonic treatment, surfactants, solvents, reaction conditions and temperature. As illustrated in Figure 3, the products FeVO_4_/AgO and ErVO_4_/AgO are composed of spherical aggregated particles, which exhibit an average size ranging from 40 to 70 nm. The FT‐IR spectrum of the pure FeVO_4_/AgO NPs and ErVO_4_/AgO nanocomposites after calcination at 500°C has been reported in Figure 4, respectively. The characterization peaks in the FeVO_4_ NP spectrum are 810 cm^−1^ (vibration of atoms in tetrahedral oxygen environment; V–O at FeVO_4_) and 539 cm^−1^ (vibration of ytterbium atoms in the octahedral oxygen environment in YbVO4 NPs and the characterization peaks in the ErVO_4_ NP spectrum are 669 cm^−1^ (vibration of O–V–O) and 536 (vibration of Er–O). Furthermore, FT‐IR was conducted to investigate the presence of specific functional groups in AgO NPs, as illustrated in the accompanying figure. The FT‐IR spectrum reveals three absorption bands at 451 cm^−1^, which can be attributed to the Ag–O bond.

### 3.2 Anthelmintic Treatment Study

#### 3.2.1 Worm Motility Assays

The viability of adult worms was assessed following 12 and 24 hours of incubation with FeVO_4_/AgO, ErVO_4_/AgO and TCBZ, respectively. The findings revealed that after 24 hours of exposure to the highest concentrations of FeVO_4_/AgO NP (5 mg/mL) and ErVO_4_/AgO NP (5.5 mg/mL), all adult *F. hepatica* specimens were killed. Additionally, after 24 hours of treatment with TCBZ at a concentration of 15 µg/mL, all adult *F. hepatica* were also killed. The results distinctly demonstrated a reduction in motility corresponding to the increasing concentrations of the four compounds. Moreover, the decline in motility observed in flukes treated with the experimental drugs was dependent on both time and concentration, as illustrated in Table 1.

**TABLE 1:** The effect of different concentrations of FeVO_4_/AgO and ErVO_4_/AgO NPs compared to TCBZ on the survival of *F. hepatica* adult worms 12 and 24 hours after being treated in the culture medium.

Test groups	Concentration (mg/mL)	Live worm		Dead worm	
	Time (hour)	12 hours	24 hours	12 hours	24 hours
FeVo4/Ago	4.5	5	1	0	4
ErVo4/Ago		5	3	0	2
FeVo4/Ago	5	2	0	3	5
ErVo4/Ago		5	1	0	5
FeVo4/Ago	5.5	1	0	4	5
ErVo4/Ago		2	0	3	5
FeVo4/Ago	6	0	0	5	5
ErVo4/Ago		0	0	5	5
	5	5	5	0	0
Triclabendazole	10	3	1	2	4
Positive control	15	2	0	3	5
	20	0	0	5	5
Negative control		5	5	0	0
Comparison between groups	*P* < 0.001				

The appropriate concentrations of TCBZ to kill different percentages of *F. hepatica* worms in 12 and 24 hours have been compared. The highest and lowest LD_50_ at 12 and 24 hours are 12.6 and 8.4, respectively. At 12 hours, only FeVo4/Ago NPs with a concentration of 6 mg/mL have maximum lethality, and, then of those, TCBZ with a concentration of 20 µg/mL has the highest lethality (Table 2).

**TABLE 2:** Comparison of lethal concentrations or LD of TCBZ in 12 and 24 hours.

Test groups	LD (mg/mL) Time (hour)	LD_10_	LD_25_	LD_50_	LD_75_	LD_90_
FeVo4/Ago	12	4.6	4.8	4.9	5.3	5.7
ErVo4/Ago		5.1	5.2	5.4	5.7	5.9
Triclabendazole (positive control)		8.6	9	12.6	16.6	18.6
FeVo4/Ago	24	3	3.4	4	4.3	4.7
ErVo4/Ago		3.8	4.2	4.7	4.9	5.2
Triclabendazole (positive control)		6.2	7.7	8.4	9.3	11.3
Comparison between groups	0.001 > *P*					

#### 3.2.2 SEM of Adult *F. hepatica*

**FIGURE 5:**
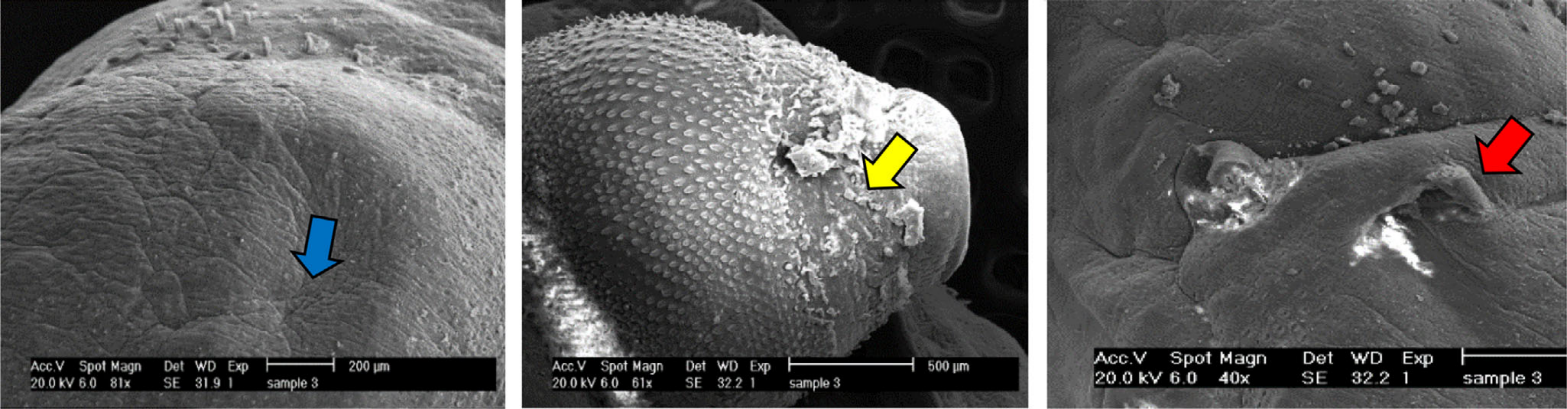
Ultrastructural changes of the adult *F. hepatica* worm after exposure to ErVO_4_/AgO NPs with a concentration of 6 mg/mL in 24 hours.

**FIGURE 6:**
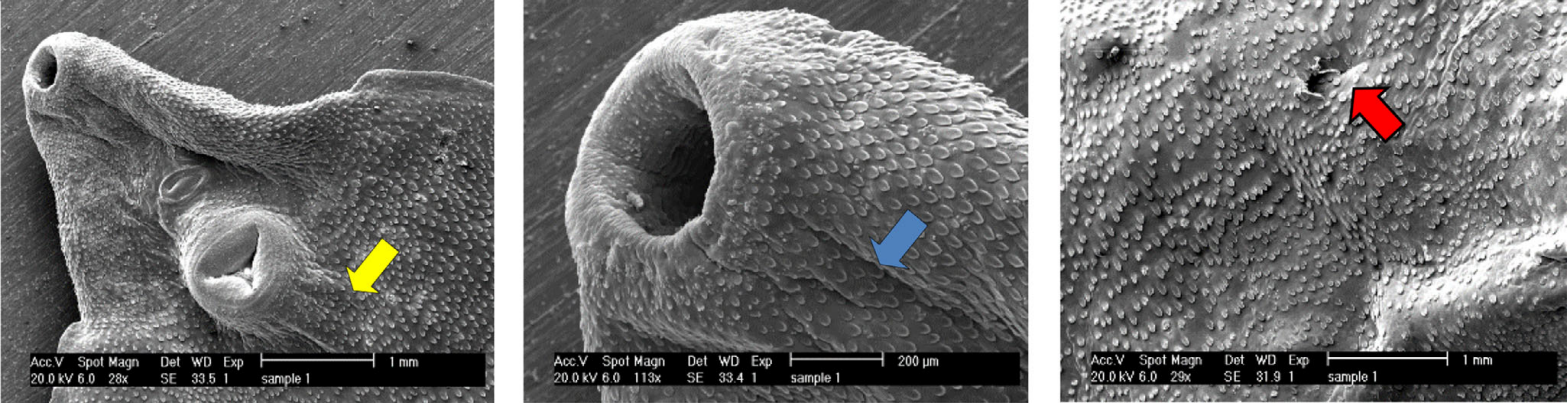
Ultrastructural changes of the adult *F. hepatica* worm after the effect of TCBZ with a concentration of 20 µg/mL in 24 hours.

**FIGURE 7:**
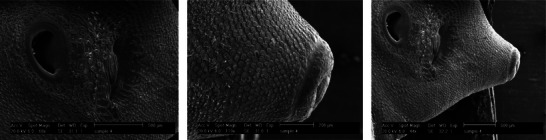
Ultrastructural changes of the adult *F. hepatica* worm in the control group after 24 hours.

The examination of the tegumental topographical alterations in *F. hepatica* liver flukes was conducted on the impacts of FeVO_4_/AgO, ErVO_4_/AgO and TCBZ, as a control group, on the surface morphology of *F. hepatica*. The control specimens, which were incubated in RPMI‐1640 culture media, exhibited normal characteristics, displaying round and smooth oral and ventral suckers, as well as an intact tegumental region surrounding the suckers. In untreated worms, the sensory papillae located at the edges and within the oral sucker, as well as the tegumental ridges network, exhibit a normal appearance, encompassing the entire body. Both the walls of the ridges and the floors of the valleys are densely populated with tegumental vesicles, which resemble spherical structures (Figure 4). In contrast, SEM of worms treated with FeVO_4_/AgO NPs (6 mg/mL) revealed that the tegument displays numerous cavities and pores on its surface. Additionally, the sensory papillae appear to be destroyed and are no longer visible. Furthermore, significant tegumental damage characterized by the destruction of prominent network structures and tegument vesicles was clearly observed (Figure 5). The extent of the damage to the tegument is such that the spins are only discernible in certain areas. SEM images of *F. hepatica* treated with ErVO_4_/AgO NPs (6 mg/mL) are presented, revealing that some sensory papillae were destroyed around the region of the oral sucker. Additionally, the images depict the absence of spins and the presence of large holes in various regions of the tegument (Figure 6). SEM images of *F. hepatica* treated with TCBZ (20 µg/mL) shown in Figure 7 illustrate the formation of pores, ruptures and shrinkage on the surface of the tegument. These findings indicate that severe tegumental damage is associated with the treatment of the worms using NPs.

All the images related to the control group show that there were no significant changes, and the tegument surface, air ducts, sensory papillae, vesicles and tegument spines were kept unchanged and swelling, wrinkling, holes, tears and blisters are not observed.

## 4 Discussion

*Fasciola hepatica* is recognized as one of the most pathogenic liver trematodes affecting sheep, goats, cattle and humans in various regions globally, and to date treatment options are limited, and no vaccine is available to control this liver fluke (Rosas‐Hostos Infantes et al. 2023). At present, the treatment for *Fasciola* relies on the administration of anthelmintic agents, which could result in the emergence of drug resistance in humans (Gandhi et al. 2019; Dermauw et al. 2021; Luis Marcos et al. 2021). Despite the widespread availability of chemical antiparasitic medications, the adverse effects associated with some of these drugs have led numerous researchers to explore alternative components. Our study evaluates two specific components, erbium vanadate/silver oxide (ErVO_4_/AgO) and iron vanadate/silver oxide (FeVO_4_/AgO) NPs, in relation to the ultrastructure of the adult liver fluke *F. hepatica*. To date, there has been no comprehensive investigation into the effects of ErVO_4_/AgO and FeVO_4_/AgO nanocomposites on the ultrastructure of *F. hepatica*. To the best of the author's knowledge, this represents the first research examining the influence of FeVO_4_/AgO and ErVO_4_/AgO on the structure and motility of adult *F. hepatica*.

Concurrently, various investigations have been undertaken regarding the impact of metal NPs on platyhelminths. The anthelmintic properties of gold and zinc oxide (ZnO) NPs against tapeworm *Raillietina* and liver amphistome, *Gigantocotyle explanatum* were examined at different concentrations. Observations made through electron microscopy revealed several alterations, including shrinkage, aggregation of the suckers, formation of holes and folds, wrinkling, indentation, rupture, tearing, and damage to both the spines and the tegument cells (Kar et al. 2014; Khan et al. 2015).

In addition, some research demonstrated the anthelmintic properties of ZnO and FeO NPs at concentrations against nematode *Toxocara vitulorum*. Notably, FeO NPs exhibited greater effectiveness compared to zinc oxide, and both types of NPs led to the mortality of all worms by elevating malondialdehyde and nitric oxide levels while reducing superoxide dismutase (SOD) activity (Dorostkar et al. 2017).

Research indicated that silver NPs lead to a decrease in the production of lipids, proteins and glycogen, which ultimately leads to the mortality of the worms and alterations in the morphology of *Haemonchus contortus* nematode eggs (Preet and Tomar 2017).

The effects of silver NPs (AgNPs) on the ultrastructural features of the adult liver trematode, *Gigantocotyle* *explanatum*, were investigated using in vitro methods. The findings indicated that the motility of the flukes treated with AgNPs was significantly diminished compared to the untreated control group. At the highest concentration, there was notable damage to the tegument, characterized by numerous blisters and a loss of sensory papillae and tubercles. Reactive oxygen species (ROS) production by flukes was observed to change, displaying a dose‐dependent rise in ROS levels in worm cells treated with AgNPs and an increase in light absorption compared to the control group. Additionally, measurements of SOD activity, an enzyme involved in oxidative processes related to ROS, revealed a significant decrease, indicating that AgNPs inhibited this enzyme's function. Furthermore, the analysis of DNA fragmentation suggested that AgNP treatment could induce apoptosis in the worms, while the level of protein carbonylation significantly rose following AgNP examination, as assessed through the protein carbonylation reaction (Rehman et al. 2019).

An in vitro study was conducted to evaluate the antiparasitic effects of silver NPs in comparison to TCBZ against *F. hepatica* eggs. The hatching percentages for experiments and its control groups clearly demonstrated that silver NPs possess the capability to eliminate eggs effectively (Gherbawy et al. 2013).

The efficacy of silver NPs as an anthelmintic agent against the gastrointestinal nematode *Haemonchus contortus* was demonstrated across various concentrations (Tomar et al. 2017).

Silver NPs have been demonstrated to exert lethal effects on the infective larvae of *Ancylostoma caninum* (L3) (Barbosa et al. 2019).

Several studies have indicated that gold and silver NPs as antiparasitic agents exert considerable effects on the blood flukes, *Schistosoma mansoni* and *S. japonicum*. Nevertheless, at higher concentrations, the cercariae not only exhibited tail loss but also became unable to infect the host. SEM images demonstrated a notable shrinkage of the cercariae (Cheng et al. 2013; Moustafa et al. 2018).

The anthelmintic properties of silver NPs against the complete immobilization microfilariae population of the tissue nematode, *Brugia malayi*, were assessed across different concentrations. Additionally, a notable inhibition of the enzyme adenosine diphosphate ribosylation factor (polymerase) was detected in microfilariae exposed to the NPs. Images obtained from an electron microscope revealed multiple perforations in the cuticle of microfilariae treated with silver NPs (Singh et al. 2012).

Several investigations showed the significant impact of silver and selenium NPs on the protoscolices of hydatid cysts, utilizing various concentrations. To assess the mortality of the parasites, a staining method with 0.1% eosin was employed. The findings indicate that both NPs possess a lethal effect on the protoscolices of hydatid cysts (Mahmoudvand et al. 2014; Lashkarizadeh et al. 2015).

AgNPs present a promising technological advancement in the fight against pathogens, due to their large surface area, liberation of silver ions (Ag+), and the generation of ROS. These factors contribute to oxidative stress and have been confirmed for their antibacterial, antiprotozoal and anthelmintic effects (Norouzi et al. 2017; Xu et al. 2020; Zhang et al. 2023). However, there is limited knowledge regarding the mechanisms by which AgNPs inactivate parasitic helminths (AbouElez et al. 2023), and the impact of AgNPs on parasites is closely associated with the influx of Ag+ and the production of ROS (Cameron et al. 2016). Helminths exposed to silver oxide NPs may experience oxidative stress, primarily due to an elevation in ROS production and the suppression of antioxidant enzymes such as SOD. This process can ultimately result in the elimination of parasites (Yu et al. 2013). Additionally, NPs that promote ROS generation also influence the function of ROS metabolic enzymes, including SOD and catalase. SOD, which serves as the principal antioxidant enzyme aiding the parasite in neutralizing free radicals generated by oxidative stress, is significantly diminished. While a slight increase in SOD levels is observed at the lowest concentrations of NPs–potentially indicating a survival mechanism of the worms to counteract the heightened ROS levels–higher concentrations of NPs appear to disrupt the antioxidant defence system (Saini et al. 2016; Zhang et al. 2023). In a normal physiological state, a specific level of ROS exists within the cell, maintained in equilibrium with the antioxidant system. Following exposure to AgNPs, cells rapidly generate significant quantities of ROS, prompting the antioxidant system to upregulate various proteins that mitigate excess ROS, including SOD, catalase and glutathione (GSH). Glutathione plays a crucial role in binding and neutralizing ROS; consequently, the antioxidant system regulated by glutathione is regarded as a vital protective mechanism for cellular survival. However, AgNPs diminish GSH levels by inhibiting the activity of GSH synthase, thereby impairing the cells' ability to effectively eliminate intracellular ROS (Zhang et al. 2023). The principal action of silver NPs involves the disruption of the fluidity and structural integrity of the parasite cell membrane. This disruption results in increased permeability and the subsequent loss of intracellular components, ultimately triggering apoptosis and effectively eliminating parasites. This process primarily occurs through the generation of ROS, which instigates oxidative stress and inflicts damage on cellular structures. Such damage can lead to apoptosis within the parasitic tissue, as well as the induction of chromosomal analysis and DNA damage (Hassan et al. 2019; Keiser et al. 2019; Zhang et al. 2023). Nanosilver possesses the ability to disrupt the cell membrane or chemically adhere to and accumulate on the cell surface, resulting in a toxic impact on the cell. AgNPs release silver ions that are harmful to certain parasites (Al‐Quraishy et al. 2020). The liberated silver ions can attach to the cell membrane of the parasite, compromising its fluidity and viability. This interaction leads to enhanced permeability and the depletion of essential intracellular components, ultimately disrupting normal cellular functions and culminating in cell death (Saha et al. 2016; Zhang et al. 2023). AgNPs induce apoptosis in cells and effectively eliminate parasites primarily through the generation of ROS (Cameron et al. 2016). The majority of intracellular stress responses are triggered by ROS‐linked toxicity, and oxidative stress is viewed as the most probable mechanism underlying AgNP‐induced cell damage. Additionally, the release of Ag+ can generate ROS within the parasite, resulting in oxidative stress and damage to cellular structures, ultimately leading to the death of the parasite cells and their eradication (Ullah et al. 2018). The disparity between the generation of ROS and their elimination of the antioxidant system can result in oxidative stress, which may lead to numerous severe consequences, including DNA fragmentation, damage to mitochondria, peroxidation of proteins and lipids and, ultimately, apoptosis (Flores‐Lopez et al. 2019). Furthermore, silver ions have the ability to interrupt ATP production and interfere with metabolic processes by harming the electron transport chain. They can also obstruct the function of crucial enzymes and metabolic pathways within the parasite, resulting in cellular damage and death (Zou et al. 2017). Currently, the precise mechanism by which AgNPs exert their effects on the parasite remains under exploration, and alternative effective methods may exist. Nevertheless, it is evident that AgNPs hold promise as a valuable asset in combating parasitic infections (Ghorbani et al. 2019).

## 5 Conclusions

The study indicated that iron vanadate/silver oxide NPs exhibit a more pronounced lethal effect on the ultrastructure of adult *F. hepatica* compared to erbium vanadate/silver oxide NPs. The findings revealed that the tegument is swollen in certain areas, the pores are distinctly visible, sensory papillae are absent and the tegument is significantly compromised, with the prominent network structure and its vesicles completely eradicated. The efficacy of both compounds is influenced by the concentration and time of the drug's effect. Additional research and development is essential to assess their potential implications for human and animal health, environmental impact, safety, stability and drug resistance.

## Author Contributions

AM performed data curtain, and formal analysis, developed the methodology and wrote the original draft. EM performed writing, review and editing. EM and HH performed data collection. All authors critically revised the manuscript and approved the final version.

## Ethics Statement

This project was found to be in accordance with the ethical principles and the national norms and standards for conducting Medical Research in Iran. The study protocol was approved by the Ethics Committee of Kashan University of Medical Sciences, Iran (approval ID: IR.KAUMS.MEDNT.REC.1398.060).

## Conflicts of Interest

The authors declare no conflicts of interest.

### Peer Review

The peer review history for this article is available at https://publons.com/publon/10.1002/vms3.70357.

## Data Availability

All data generated or analysed during this study are included in this published article.
